# The art of sharing: From research to outreach in the social media era

**DOI:** 10.1113/EP092887

**Published:** 2025-07-09

**Authors:** Milan Mohammad, Abdullahi A. Mohamed, Jari Dahmen, Jonathan J. Bjerre‐Bastos, Andreas Pihl

**Affiliations:** ^1^ Centre for Physical Activity Research Copenhagen University Hospital – Rigshospitalet Copenhagen Denmark; ^2^ Department of Cellular and Molecular Medicine, Faculty of Health and Medical Sciences University of Copenhagen Copenhagen Denmark; ^3^ Department of Cardiology Herlev‐Gentofte University Hospital Copenhagen Denmark; ^4^ Department of Orthopedic Surgery and Sports Medicine, Amsterdam Movement Sciences, Amsterdam UMC Location AMC University of Amsterdam Amsterdam The Netherlands; ^5^ Academic Center for Evidence‐based Sports Medicine (ACES), Amsterdam UMC Amsterdam The Netherlands; ^6^ Amsterdam Collaboration for Health & Safety in Sports (ACHSS) International Olympic Committee (IOC) Research Center, Amsterdam UMC Amsterdam The Netherlands; ^7^ Department of Orthopedic Surgery Bispebjerg Hospital Copenhagen Denmark; ^8^ Roche Diagnostics Copenhagen Denmark

1

Historically, scientific knowledge has been disseminated through carefully curated channels: from the scholarly exchanges of the Royal Society in the 17th century to the peer‐reviewed journals of today. However, the digital era has reshaped traditional dissemination, raising questions about how scientific findings reach audiences and how credibility is maintained in a time of instant information exchange. Although the h‐index, impact factor and other citation‐based metrics have long served as the primary indicators of scholarly influence, they come with limitations and may not necessarily reveal the broader societal impact of research (Garfield, 2006; Hirsch, 2005). As such, the impact factor is criticised for over‐representing a small subset of highly cited articles, and the h‐index for its lack of field normalizations and disregard for author order (Hicks et al., 2015). Therefore, physiology in particular, rooted in empirical observation and methodological rigour, has long grappled with how best to measure the significance of discoveries beyond citations (Berg et al., 2025). In this editorial, we explore how academic evaluation is moving beyond traditional citation metrics towards broader measures, such as altmetrics and social media indicators. Although these measures expand research reach, they also challenge rigour, equity and ethics. This requires clear standards, thorough validation and supportive institutional policies.

Back in the 18th and 19th centuries, physiological findings were shared through salons, learned societies and handwritten correspondences, often awaiting formal critique for months or years (McClellan III, 1993; Worboys, 2011). These forums operated almost like a closed club, in stark contrast to today's instantaneous digital ecosystem, where new results can be broadcast globally in seconds and are immediately subject to public scrutiny and debate (Vineis, 2024). By the mid‐20th century, Eugene Garfield and Irving H. Sher introduced the concept of the impact factor, which formalized the notion of ‘who is reading whom’, shaping an era in which publication venues and citation counts became central to a scientist's reputation (Garfield & Sher, 1963). Although the impact factor laid the groundwork, a range of other citation‐based indicators soon emerged to capture different dimensions of scholarly output. Traditional citation‐based metrics, such as the h‐index and journal impact factor, provide snapshots of academic productivity, but do not necessarily reveal how (or whether) a discovery guides clinical practice, informs policy decisions or engages public interest (Bornmann & Marx, 2016; Grant, 1999; López‐Cózar et al., 2014; Raftery et al., 2016). The h‐index, introduced in 2005 by Hirsch, was intended to capture both productivity and citation impact (Hirsch, 2005). However, its structure incentivises strategic behaviours that can distort academic evaluation. Researchers may engage in excessive self‐citation or form citation cartels, which are agreements among groups of authors to cite each other's work systematically, all to boost their scores artificially (Bi, 2023; Fong & Wilhite, 2017). These practices distort genuine scholarly impact.

This is not the only issue. Discrepancies among citation databases (e.g., Web of Science, Scopus, Google Scholar) stem from their differing coverage policies and indexing practices. For instance, Google Scholar includes a broader array of sources, encompassing theses, books, conference papers and non‐English materials, many of which are excluded by the more selective Web of Science and Scopus. This divergence leads to significant variations in citation counts and impact metrics for the same publication, thereby undermining the consistency and reliability of citation‐based evaluations across disciplines (Martín‐Martín et al., 2018).

Moreover, the journal impact factor, originally designed to help libraries assess journal quality, has also been misused as a proxy for individual researcher performance, despite criticisms of its opacity and susceptibility to editorial manipulation. Editors may engage in practices such as encouraging authors to cite articles from the same journal, preferentially publishing review articles that typically attract more citations or strategically timing publications to maximize citation counts within the calculation window of the impact factor (López‐Cózar et al., 2014). Although both the impact factor and the h‐index have limitations, improvements have been proposed without being implemented fully. This includes normalizing journal citation rates against field‐specific benchmarks to reduce disciplinary bias (Hutchins et al., 2016) and adopting a fractional h‐index that allocates citations proportionally among co‐authors to mitigate the effects of hyper‐authorship and self‐citation (Koltun & Hafner, 2021).

By recognizing these limitations, alternative models have emerged to capture better the significance of research beyond academia. Societal impact factors have been proposed to evaluate how findings contribute to policy, healthcare and industry (Kalucy et al., 2009; Penfield et al., 2014). More recently, altmetrics have gained traction as a tool for tracking online engagement through social media mentions, news coverage, blog posts and other public discussions, often preceding formal citations (Eysenbach, 2011; Kousha & Thelwall, 2016; Priem et al., 2011; Weller, 2015). Unlike traditional citations, which accumulate slowly, altmetrics provide near‐instant feedback, offering insights into the reach of a study and its resonance with diverse audiences (Warren et al., 2017). A common example is the Altmetric Attention Score, a weighted composite that reflects the quantity and quality of attention an article receives across multiple platforms, such as Twitter, Facebook, policy documents and major news outlets. Although higher scores indicate broader dissemination and engagement, they can be skewed by media sensationalism or topical popularity rather than genuine scholarly merit. Strategic amplification by authors with extensive online networks or institutional public relations teams through large follower cohorts, coordinated sharing or paid bot activity can artificially elevate scores and decouple apparent attention from true academic impact (Haustein et al., 2016). The metric has also been widely criticised for its unclear weighting scheme and lack of empirical justification (Gumpenberger et al., 2016). Yet, its integration as live badges across databases, repositories and journals has made it a widespread indicator of research visibility (Elmore, 2018). Although altmetrics face these challenges, they might still represent a valuable supplement to conventional metrics, shifting the focus from purely academic impact to broader societal relevance.

Given that altmetric scores draw on activity across social media channels, it is crucial to examine how those channels shape visibility and engagement. As such, the increasing use of social media in scientific discourse has changed the way research is shared. Platforms such as X (formerly Twitter), Bluesky, LinkedIn, ResearchGate, Facebook, Reddit, TikTok and Instagram now enable researchers to engage with colleagues and the public in unprecedented ways. Some journals include comment sections to foster debate, and publishers now encourage or even require graphical abstracts, tweetable highlights and other brief summaries, viewing them as integral to the broader dissemination process (Bik & Goldstein, 2013). Additionally, podcasts are gaining traction as a tool for scientific discourse (Persohn & Branson, 2025). It is estimated that a large proportion of the public now obtain their news from social media, and surveys suggest that a growing number of scientists also use these platforms to follow discoveries and discuss research (Brossard, 2013; Smailhodzic et al., 2016). The rapid exchange of scientific information on social media partly explains the rise of altmetrics, because altmetrics draw on digital traces such as tweets, shares and bookmarks to quantify in real time how research circulates across platforms. Moreover, physiologists who have shared preliminary data on social media have observed spikes in readership, discussion and, eventually, citations (Eysenbach, 2011; Fraser et al., 2021; Pulido et al., 2018). Research mentioned on social media often receives additional citations, such as video citations (Shaikh et al., 2023), and some data suggest a correlation between social media visibility and long‐term scholarly impact (Carvalho et al., 2025). Critics caution that ‘likes’ and ‘retweets’ do not equate to scholarly merit and that altmetrics, being primarily quantitative, might not reflect the quality of engagement. Nevertheless, altmetrics might serve as a useful supplement for measuring broader resonance (Patthi et al., 2017). Although it took years for the ideas of Claude Bernard, considered one of the fathers of physiology as an experimentally based science, to gain acceptance of the idea of ‘milieu intérieur’ in the 19th century, later expanded upon through the concept of homeostasis by Walter B. Cannon and Homer W. Smith, one might wonder whether ‘live‐tweets’ from the laboratory might have fast‐tracked results or at least sparked a lively online discussion about methodological rigour (Bernard et al., 1878; Cannon, 1926; Smith Homer William, 1959).

Nonetheless, the relationship between social media visibility and scientific credibility remains complex. Social media platforms prioritize content that is engaging, concise and shareable, which are qualities that do not necessarily align with rigorous scientific inquiry. Studies indicate that research framed in more dramatic or provocative terms tends to receive higher altmetric scores, regardless of its methodological quality (West & Bergstrom, 2021). This phenomenon creates a curation bias, whereby easily digestible narratives attract disproportionate attention, whereas more nuanced or technically complex research struggles to gain traction (Carvalho et al., 2025). Furthermore, the inherent structure of social media amplifies the voices of high‐profile researchers and institutions, reinforcing disparities in academic visibility and influence (West & Bergstrom, 2021).

A fundamental question arises: When researchers share their findings online, who is engaging? The intended audience might be fellow scientists, but much of the discussion often involves a broader and more heterogeneous group, including journalists, policymakers, lay audiences or, at the very least, non‐scientists. On the one hand, broadening the reach of scientific findings can promote public engagement and literacy (Retzbach & Maier, 2015; Sinatra & Hofer, 2016). On the other hand, oversimplified or decontextualized interpretations might distort the message (Aïmeur et al., 2023). The politicization of science adds another layer of complexity. Findings are often interpreted through ideological lenses, and public acceptance can be dictated by political affiliation (Drummond & Tipton, 2024; Lewandowsky et al., 2013). During the coronavirus 2019 (COVID‐19) pandemic, debates over vaccine efficacy and public health measures became polarized (Wagner & Eberl, 2024). Studies suggest that misinformation spreads six times faster than factual content, making it easier for false or misleading interpretations to gain traction (Vosoughi et al., 2018). One key contributor to this ecosystem of error is the rise of predatory journals, which further undermine scientific credibility by exploiting the same attention dynamics of social media to promote unverified or low‐quality research (Azizova & Okhunov, 2023; Teixeira da Silva & Huang, 2024). Unlike established society journals, which adhere to stringent peer‐review processes, predatory publishers often prioritize sensationalism over scientific rigour, making them well suited to the engagement‐driven economy of social media. This can lead to the unintended legitimization of questionable research, particularly among non‐expert audiences, who might struggle to differentiate between reputable and unreliable sources when seeing studies shared on social media (Daly et al., 2024; Teixeira da Silva, 2022). Additionally, artificial intelligence‐generated fake studies, manipulated datasets and fabricated peer reviews are becoming increasingly difficult to detect, further blurring the line between legitimate and misleading research, especially when labelled on social media as peer‐reviewed studies from a credible publisher (Chaka, 2024; Májovský et al., 2023). Without robust verification mechanisms, the risk of scientific distortion increases, making it ever more difficult for researchers and the public to distinguish fact from fiction. As such, researchers have increasingly taken the matter into their own hands, using platforms such as PubPeer for post‐publication critique and non‐profit initiatives such as Retraction Watch to flag and review retracted publications in real time, exposing patterns of misconduct and enhancing transparency. Even these community‐driven checks cannot shield individuals from the personal risks of online engagement.

Beyond misinformation, researchers who engage in public discourse, particularly those discussing ‘controversial’ topics, can face harassment, threats and doxing (Royan et al., 2023). Women and scientists from underrepresented backgrounds are disproportionately affected, with many reporting experiences of intimidation aimed at silencing their voices (Egelhofer et al., 2024), which is a chilling effect that risks deterring valuable scientific contributions from public view. Studies show that witnessing scientists being attacked online reduces public confidence in their work, leading to scepticism not only towards the targeted researcher but towards the scientific community as a whole (Egelhofer et al., 2024). This could also lead other researchers to be reluctant about sharing their studies on social media and promoting them, in fear of being attacked or ‘going viral’.

Given these challenges, there is a pressing need for structured guidelines on responsible social media use. A recent consensus report from the Italian Society of Anesthesia, Analgesia, Resuscitation and Intensive Care (SIAARTI) emphasizes the importance of ethical dissemination, urging researchers to uphold transparency and accuracy in online communication (Cortegiani et al., 2024). Institutional guidelines may vary, but key recommendations mainly include ensuring that peer‐reviewed results are clearly distinguished from preliminary data, that conflicts of interest and funding sources are disclosed in every communication, that patient or sensitive information is never revealed, and that any errors are corrected promptly and publicly. As platforms and norms continue to evolve, these ethical standards must be revisited and adapted such that scientists can harness the reach of social media without compromising integrity. Academic institutions should also integrate digital science communication training into PhD programmes, equipping early‐career researchers with the skills to navigate public‐facing engagement responsibly. This includes teaching best practices for communicating complex physiological data to diverse audiences, emphasizing ethical considerations and providing strategies for maintaining scientific rigour across different social media platforms. Additionally, universities and professional societies should implement clear policies to protect scientists from online harassment, providing support structures for those facing digital abuse. Finally, the role of the peer review must not be underestimated, not only for validating research but also for protecting scientists in an era of digital scrutiny. By ensuring that findings undergo expert evaluation before public discussion, peer review acts as a safeguard against misinformation, misrepresentation and the reputational risks associated with premature or controversial findings (Tennant et al., 2017). Although the peer review model is challenged by the shortage of referees, maintaining quality peer review by trusted journals sustains the credibility of research (Berg et al., 2024; Ellaway, 2024).

Despite these challenges, the use of social media holds great potential for expanding one's research. Large‐scale trials have used Twitter handles and updates to inspire site participation, recruit participants and maintain real‐time momentum (Chu & Snider, 2013; Darmawan et al., 2020). Participant recruitment, in particular, can benefit from the extensive reach of social media (Feyz et al., 2020). Traditional strategies, such as partnering with community organizations or distributing brochures in clinics, remain crucial, but online approaches can also draw individuals from remote or underrepresented populations. In addition, online recruitment enables participants to follow the progress of the study and stay informed about the final publication, ultimately fostering a sense of inclusion in the research. However, relying solely on social media can skew sampling. Thus, physiologists should blend online efforts with established offline methods to safeguard data integrity and ensure demographic balance.

As the scope of scientific communication expands, our strategies for preserving rigour, transparency and ethical responsibility must follow suit. Digital platforms offer unprecedented opportunities but also introduce new risks that must be managed carefully. One might imagine Santorio Santorio, who weighed himself daily to study insensible perspiration, enthusiastically ‘live tweeting’ from his laboratory if the technology had existed (Santorio, 1614). The challenge now is not only to share findings widely on social media but to do so responsibly and thoughtfully, with an unwavering commitment to scientific integrity. The spirit of sharing data to expand knowledge is as crucial today as it was in the dawn of experimental physiology.

To conclude, although citation‐based metrics remain dominant in academia and continue to shape the careers and advancement of researchers, they fail to capture the full value of scientific work and researcher contributions. Scientific dissemination should not be driven by career incentives alone. Research must inform, educate and influence clinicians, policymakers and the public rather than simply boosting citation counts. Although it might be more pragmatic to judge publications and researchers by numbers, a comprehensive evaluation should include a qualitative perspective, considering factors such as societal benefit, policy influence and educational reach alongside traditional metrics and using emerging digital indicators of online engagement. To turn these principles into practice, more institutions are now embracing responsible‐metrics frameworks, urging that citation counts be complemented by measures of data sharing, reproducibility and real‐world influence rather than used in isolation. Moreover, narrative curriculum vitae formats allow researchers to contextualize their contributions with concrete examples of outreach, collaboration and impact. Achieving the perfect balance will require a diverse toolkit of quantitative and qualitative measures, together with training in science communication and outreach. This is a formidable but essential endeavour, both for career development and for capturing the true impact of future physiology studies.

## AUTHOR CONTRIBUTIONS

Milan Mohammad: Conception, first draft, revisions. Abdullahi A. Mohamed: Conception, revisions. Jari Dahmen: Conception, revisions. Jonathan J. Bjerre‐Bastos: Conception, revisions. Andreas Pihl: Conception, first draft, revisions. All authors approved the final version of the manuscript and agree to be accountable for all aspects of the work in ensuring that questions related to the accuracy or integrity of any part of the work are appropriately investigated and resolved. All persons designated as authors qualify for authorship, and all those who qualify for authorship are listed.

## CONFLICT OF INTEREST

The authors declare no conflicts of interest.

## FUNDING INFORMATION

None.
